# Effect of ultrahigh temperature treatment on qualities of watermelon juice

**DOI:** 10.1002/fsn3.593

**Published:** 2018-02-06

**Authors:** Yubin Wang, Xiaofei Guo, Yue Ma, Xiaoyan Zhao, Chao Zhang

**Affiliations:** ^1^ Beijing Vegetable Research Center Beijing Academy of Agriculture and Forestry Sciences Beijing China; ^2^ Beijing Key Laboratory of Fruits and Vegetable Storage and Processing Ministry of Agriculture Beijing China; ^3^ Key Laboratory of Vegetable Postharvest Processing Ministry of Agriculture Beijing China; ^4^ Key Laboratory of Biology and Genetic Improvement of Horticultural Crops (North China) Ministry of Agriculture Beijing China

**Keywords:** (*E,Z*)‐2,6‐nonadienal, alcohol dehydrogenase, microbial colonies, polyphenoloxidase, ultrahigh temperature, watermelon

## Abstract

The watermelon juice was pasteurized by the ultrahigh temperature (UHT) treatment, and the qualities of the pasteurized juice were compared to screen the optimal UHT. The UHT of 120 and 135°C inactivated the microbial colonies and maintained the original color of the watermelon juice. The temperature of 120 and 135°C was also maintained the phenolic content by reducing the polyphenoloxidase activity. Moreover, the C9 aldehydes, especially the (*E*,*Z*)‐2,6‐nonadienal, presented the main aroma of the watermelon juice. The C9 aldehydes were formed as the results of the heat reduction and enzymatic metabolism. The temperature of 120 and 135°C reduced the alcohol dehydrogenase activity and well maintained the C9 aldehyde content of the watermelon juice. Hence, the temperature of 120°C of the UHT treatment was the optimal temperature for the production of the watermelon juice.

## 1 INTRODUCTION

Ultrahigh temperature (UHT) technique heats the feed at a high temperature in a short time, keeping the safety, and the nutrition of the feed (Charles‐Rodríguez, Nevárez‐Moorillón, Zhang, & Ortega‐Rivas, 2007). At the beginning, the UHT technique was mainly used in the milk production, maintaining the safety, and extending the shelf life of the fresh milk. Currently, the UHT technique also inactivates the microbial colonies of the watermelon juice (Zhang, Wang, Ma, Zhao, & Li, 2015), orange juice (Walkling‐Ribeiro, Noci, Cronin, Lyng, & Morgan, 2009), cucumber juice (Liu, Zhang, Zhao, Wang, & Liao, 2016), and sugarcane juice (Jittanit, Wiriyaputtipong, Charoenpornworanam, & Songsermpong, 2011). The temperature is the key factor of the UHT treatment. A higher temperature of the UHT treatment guarantees the safety of the juice (Charles‐Rodríguez et al., 2007). However, the higher temperature of the UHT treatment leads to the degradation of the color, aroma, and nutritional quality of the juice (Tiwari, O'Donnell, Muthukumarappan, & Cullen, 2009). For instants, the UHT treatment reduces the ascorbic acid content of the orange juice (Tiwari et al., 2009), and the phenolics content of apricot nectars significantly (Huang et al., 2013). Hence, it was important to find the optimal temperature to guarantee the safety of the juice as well as its qualities.

Watermelon juice is sensitive to the heat, oxygen, light, ion, etc. (Aguiló‐Aguayo, Montero‐Calderón, Soliva‐Fortuny, & Martín‐Belloso, 2010; Feng, Ghafoor, Seo, Yang, & Park, 2013; Liu, Wang, Zhang, et al., 2014; Zhang et al., 2011). In previous studies, our results proved that the UHT treatment was effective to inactivate the microbial colonies of the watermelon juice (Zhang et al., 2015). In this study, the temperature of the UHT treatment was further explored to find the balance of the safety and qualities of the watermelon juice. Moreover, the key enzymes related to the formation of the aroma were discussed to give a deeper insight into the qualities of the watermelon juice.

## 2 MATERIAL AND METHODS

### 2.1 Processing of the watermelon juice

The mature watermelon (*Citrullus lanatus* var. Jingxin No.3) was purchased from a local fruit market. The fruits were round with regular stripes and weighted about 3–4 kg per fruit. The flesh of the fruits was red and crisp with a soluble solid content (SSC) of 11.5%–13.5%.

The fruits were peeled and squeezed in a Philips juicer after stored at 4°C for 24 hr (HR1861, Philips Co. Beijing, China). The juice was mixed with complex food additive including carboxymethylcellulose sodium (0.2%), ascorbic acid (0.025%), xanthan gum (0.05%), ethylene diamine tetraacetic acid (0.01%), carminum (0.005%), and sodium pyrophosphate (0.1%). The mixture was fully stirred, and was adjusted to the pH 4.10 with the citric acid, and to the SSC of 8.0 with the high‐fructose corn sirup (4502504‐01, Fresh Juice Industry [Kunshan] Co. Ltd.). And then, the formulated juice was homogenized at the pressure of 50 MPa (NS101L2K, GEA Niro Soavi S.p.A., Parma, Italy), which was nominated as the Unpasteurized. The Unpasteurized juice was pasteurized by UHT treatment at the temperature of 110, 120, and 135°C for 2 s, which was nominated as the UHT110, UHT120, and UHT135, respectively. The pasteurized juice was sterile filled in the 300 ml PET bottles.

### 2.2 Total microbial colonies

The sample was serially diluted, plated in total count agar for total flora counts. The plates were incubated at 30°C for 48 hr, and the total microbial colonies were counted manually.

### 2.3 Determination of color and SSC

Color of the sample was measured in the reflectance mode for six times at 23°C (Chromameter CR‐300, Minolta, Japan). Color L*, a*, and b* value were measured and the total color difference (Δ*E*) was calculated by Equation (1).

(1) $$\Delta E={\left[{\left(L-L_{0}\right)}^{2}+{\left(a-a_{0}\right)}^{2}+{\left(b-b_{0}\right)}^{2}\right]}^{1/2}$$

where Δ*E* is the total color difference between a sample and the control, *L* and *L*_0_ are the lightness of a sample and the control, respectively; *a* and *a*_0_ are the redness of a sample and the control, respectively; *b* and *b*_0_ are the yellowness of a sample and the control, respectively.

The SSC was recorded by a pocket refractometer (Pal‐α, ATAGO, Japan) with distilled water as a blank.

### 2.4 Determination of phenolics content

The phenolic content was measured using Folin–Ciocalteu reagent (Yu, Perret, Harris, Wilson, & Haley, 2003). In brief, the reaction mixture contained 50 μl of the sample, 250 μl of Folin–Ciocalteu reagent, 0.75 ml of 20% sodium carbonate, and 3 ml of pure water. After the inoculation at 25°C for 2 hr, the absorbance at 765 nm was measured with the reaction mixture without sample as the control. The result is expressed as the gallic acid equivalent.

### 2.5 Measurement of polyphenoloxidase, alcohol dehydrogenase, and lipoxygenase activity

The enzymatic extract was prepared by the watermelon juice followed our recent methods (Zhang et al., 2011). Specifically, aliquot of 25 ml juice was stirred with 100 ml of the phosphate buffer (pH 6.0), 100 μl of Triton X‐100, and 3 g of polyvinylpyrrolidon. The mixture was magnetic stirred for 1 hr in the ice bath, and centigrade at 8,603 g for 15 min at 4°C. The supernatant was collected as the enzymatic extract for the following measurements, respectively.

PPO (polyphenol oxidase) activity was measured by a Shimadzu spectrophotometer (Ultraviolet‐1800, Shimadzu Corporation, Kyoto, Japan). Briefly, 100 μl of enzymatic extract was added to 1 ml substrate (0.1 mol/L catechol in McIlvaine buffer, pH 5.0), and the increase in absorbance at 400 nm at 25°C was recorded automatically for 30 min. One unit of PPO activity was defined as the change in absorbance of 0.01/min at 420 nm, which was calculated from the linear part of the curve.

The ADH (alcohol dehydrogenase) activity was estimated by a photometric method with the p‐nitrosodimethylaniline as a substrate. The reaction mixture (2 ml) contained serum (0.1 ml), 1.8 ml of a 26 μmol/L solution of substrate in 0.1 mol/L of sodium phosphate buffer, pH 8.5 and 0.1 ml of enzymatic extract containing 0.25 mol/L n‐butanol and 5 mmol/L NAD. The reduction in p‐nitrosodimethylaniline was monitored at 440 nm on a Shimadzu spectrophotometer (Ultraviolet‐1800, Shimadzu Corporation, Kyoto, Japan).

The LOX (lipoxygenase) activity was determined by continuously monitoring the formation of conjugated dienes from linoleic acid. The substrate consisted of 10 μl of linoleic acid, 4 ml of H_2_O, 1 ml of 0.12 N NaOH, and 5 ml of Tween 20. The enzymatic extract was shaken and diluted up to 25 ml with pure water. The diluted enzymatic extract of 100 μl was pipetted in a quartz with 2.7 ml of 0.2 mol/L phosphate buffer (pH 6.5) and 40 μl of the substrate. The reaction started by adding the diluted enzymatic extract, and the absorbance was measured using a spectrophotometer (Ultraviolet‐1800, Shimadzu Corporation, Kyoto, Japan) at 234 nm for 2 min at 22°C. A blank was prepared with 0.5 ml distilled water and 3.0 ml substrate solution. The activity was determined from the slope of the linear portion of the curve. One unit of LOX activity was defined as a change of 0.001 units of absorbance per minute and per milliliter of enzymatic extract.

### 2.6 Electronic nose analysis

The aroma of the samples was compared by the electronic nose (PEN2, Airsense Analytics GmbH, Schwerin, Germany). The electronic nose was turned on for 30 min and flushed the testing system for 180 s. The sample of 2 ml was put in the testing tube. And then, the electronic sensor was put into the testing tube to collect the results for 60 s.

### 2.7 GC‐MS analysis and identification of volatile compounds

An aliquot of 10 ml of the sample was put into a glass vessel. The glass vessel was placed in a water bath at 30°C. The headspace was purged with a constant flow of nitrogen at 20 ml/min for 10 min. The volatile compounds were trapped in a glass capillary tube (3 mm internal diameter) containing 100 mg of Tenax^®^ adsorbent. The Tenax^®^ trap was then disconnected from the system and placed in a Chrompack TCT/PTI 400 injector.

The volatiles of the sample were performed on an Agilent 6890 gas chromatograph coupled to an Agilent 5973I mass selective detector (Agilent Technologies, Palo Alto, CA). The volatile compounds were separated on a DB‐Wax column (30 m × 0.25 mm i.d., 0.25 μm film thickness, Agilent Technologies). The injection was performed in splitless mode (0.7 mm splitless inlet liner, Supelco) and injector temperature was 220°C. The purge valve was opened at 0.5 min at a 50 ml/min flow rate. Helium (99.99%) was used as the carrier gas with a constant starting flow rate at 0.7 ml/min. The oven temperature was programmed as follows: 35°C for 1 min, 5°C/min to 100°C, and 20°C/min to a final temperature of 250°C with a final holding time of 5 min. The detector was fitted with an electron impact ionization source set at 230°C. The quadrupole temperature was set to 150°C, and the transfer line temperature was kept at 250°C. The solvent delay was set to 3 min. Total ion chromatograms were collected scanning from m/z 30 to 150 at a rate of 3.06 scans/s.

Volatile compounds were identified by comparison of their mass spectra and retention times with those of authentic standards, or by comparison of Kovats( retention indexes and mass spectrum, with those reported in the NIST Mass Spectral Search Program (version 2.0a) with <80% as a cutoff to match compounds. The K.I.s were calculated from the retention times of C6–C40 n‐alkanes.

The aroma vitality of a volatile was the ratio of the volatile content in its threshold.

### 2.8 Statistical analysis

Analysis of variance (ANOVA) was used to compare mean differences of the results. If the differences in mean existed, multiple comparisons were performed using Duncan's multiple range test. All analysis was conducted using SPSS for Window Version 19. All experiments were carried out in triplicates or more.

## 3 RESULTS AND DISCUSSION

### 3.1 Effect of UHT temperature on the total microbial colonies of watermelon juice

**Figure 1:**
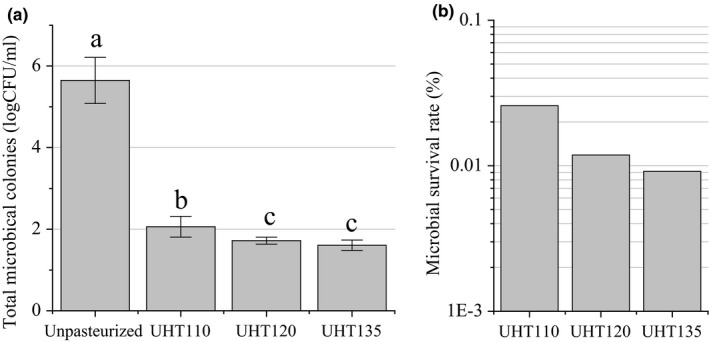
Effect of UHT on the total microbial colonies (a) and microbial survival rate (b) of the watermelon juice. Data are means ± standard deviation (*n* ≥ 3). Means with different letter represent a significant difference (*p *<* *.05)

The effect of the UHT on the total microbial colonies and microbial survival rate of the watermelon juice is shown in Figure 1. The microbial survival rate of the UHT135 was lower than 0.01% (four order), while that of the UHT110 and UHT 120 was lower than 0.1% (three order) and higher than 0.01% (four order). Hence, the temperature of 110, 120, and 135°C of the UHT treatment reduced the total microbial colonies effectively.

The total microbial colonies of the UHT135 were statistically similar to that of the UHT120, but lower than that of the UHT110. Hence, the temperature of 120 and 135°C was better to inactivate the microbial colonies. The total aerobic bacteria of the cucumber juice were reduced by 3.5 log CFU/ml pasteurized at 110°C for 8.6 s, being similar our watermelon juice. Being more efficiency than our UHT treatment, the aerobic bacterial counts in orange juice are lower than 2.0 CFU/ml only after the pasteurization at 94°C for 26 s (Walkling‐Ribeiro et al., 2009). This efficiency was resulted from the low acidity of the orange juice.

### 3.2 Effect of UHT on color and SSC of watermelon juice

**Figure 2:**
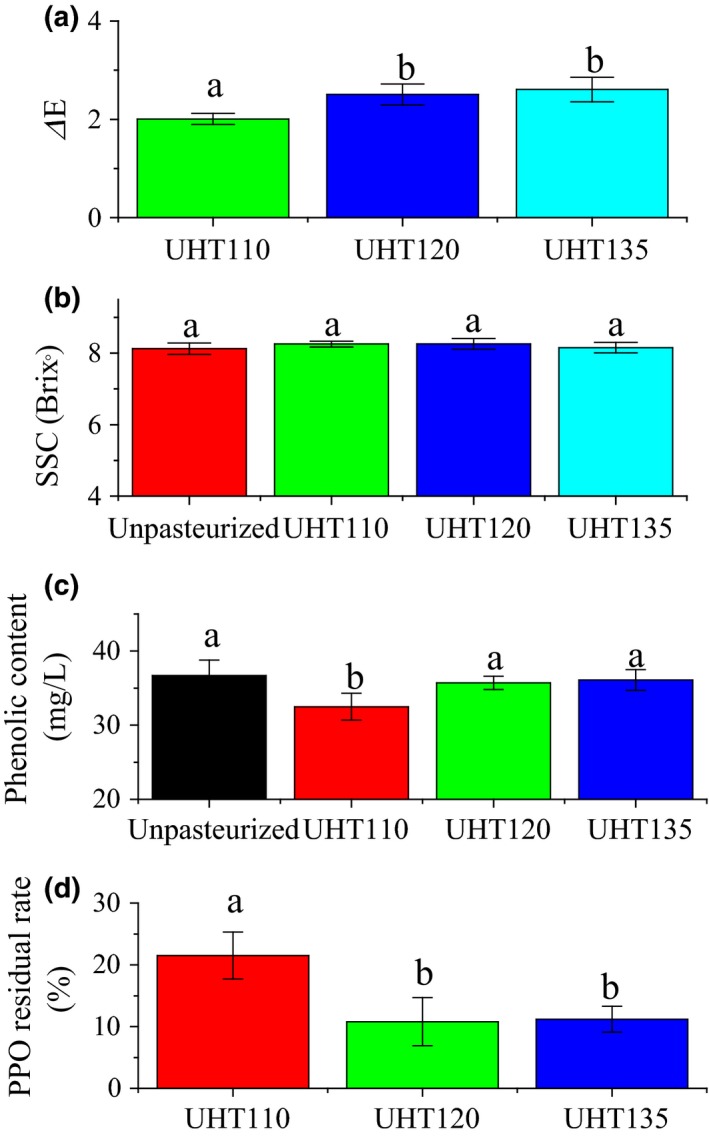
Effect of UHT on the color difference (*Δ*E) (a), SSC (b), phenolic content (c), and PPO residual rate (d) of the watermelon juice. SSC, soluble solid content; PPO, polyphenol oxidase. Data are means ± standard deviation (*n* ≥ 3). Means with different letter represent a significant difference (*p *<* *.05)

The effect of UHT on the color and SSC of the watermelon juice is shown in Figure 2. The Δ*E* of the UHT110 was significantly lower than that of the UHT120 and UHT135. Consequently, a lower temperature led a less color difference. Remarkably, each Δ*E* was lower than 6.0 when the color difference can be disguised visually by a normal person. Hence, the UHT treatment did not lead to a significant color difference in the watermelon juice. Being consistent to our results, the Δ*E* of the cucumber juice, mango nectars, and apricot nectars was only 2.99, 1.93, and 2.21 after pasteurized at 110°C for 8.6 s. (Huang et al., 2013; Liu, Wang, Li, Bi, & Liao, 2014; Liu et al., 2016), respectively, and that of the orange juice was 3.02 after pasteurized at 94°C for 26 s. (Huang et al., 2013).

The SSC of the pasteurized watermelon juice was not influenced by the temperature of the UHT treatment. Interestingly, the SSC of the Unpasteurized watermelon juice was 8.0°. The UHT treatment enhanced the SSC of the watermelon juice. The similar results were also reported in cucumber juice (Liu et al., 2016) and orange juice (Walkling‐Ribeiro et al., 2009).

### 3.3 Effect of UHT on phenolics content of watermelon juice

The phenolic content of the watermelon juice is shown in Figure 2. Usually, the phenolic content will be reduced by the heat‐induced degradation (Lee, Durst, & Wrolstad, 2002; Rossi et al., 2003). The phenolic content of the Unpasteurized was similar to that of the UHT120 and UHT135, but higher than that of the UHT110. The UHT treatment did not lead to a heat‐induced degradation of the phenolic compounds in the watermelon juice.

The phenolic compounds are regulated by a series of enzymatic reaction (Treutter, 2001). Among the reactions, PPO catalyses the o‐hydroxylation of phenol molecules to o‐diphenols, and also further catalyses the oxidation of o‐diphenols to produce o‐quinones. Consequently, a higher PPO activity will reduce the phenolic content (Gawlik‐Dziki, Złotek, & Świeca, 2008). The effect of UHT on the PPO residual rate of the watermelon juice was compared in Figure 2. The optimal temperature of the PPO is 35–40°C, and the temperature higher than 60°C reduces the PPO activity (Gawlik‐Dziki et al., 2008). The PPO residual rate of the UHT110 was about two times of that of the UHT120 and UHT135. The reduction in the PPO activity will reduce the oxidation of phenolic content (Rossi et al., 2003). Consequently, the phenolic content was mainly influenced by the reduction in the PPO activity rather than the heat‐induced degradation. Therefore, the temperature of 120 and 135°C well maintained the phenolic content by reducing the PPO activity.

### 3.4 Effect of UHT on volatiles of the watermelon juice

**Figure 3:**
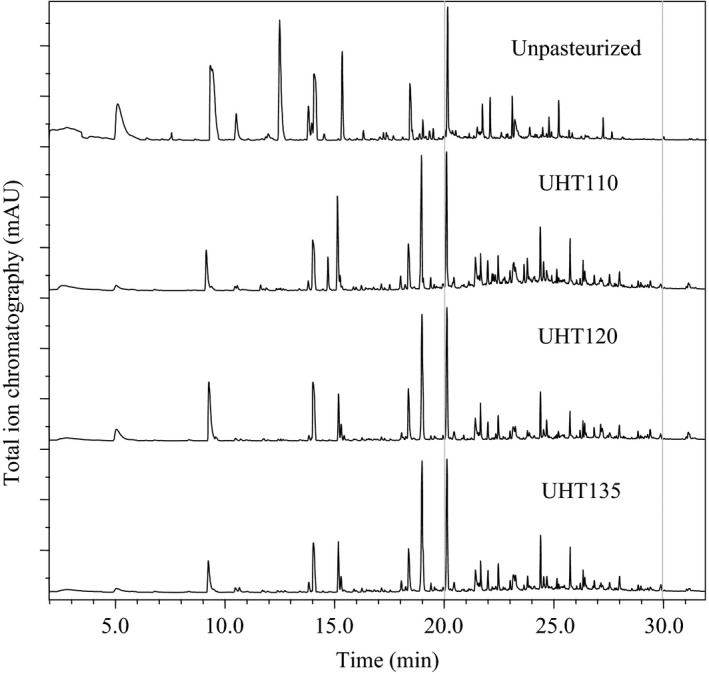
Total ion chromatography profiles of the watermelon juice

The total ion chromatography profiles of the watermelon juice are shown in Figure 3. Total 26, 22, 24, and 21 volatiles were identified in the Unpasteurized, UHT110, UHT120, and UHT135, respectively. The volatiles were further classified as the alcohol, aldehyde, ketone, ester, and the others (Table 1). The aldehyde was the main component in each watermelon juice, which constituted 71.7%, 58.9%, 53.2%, and 64.8% in the Unpasteurized, UHT110, UHT120, and UHT135, respectively.

**Table 1:** Volatiles of the watermelon juice

Type	Volatiles	Unpasteurized (%)	UHT110 (%)	UHT120 (%)	UHT135 (%)
Alcohol	(*E*)‐2‐nonenol	4.40 ± 0.24^a^a	3.63 ± 0.37 b	3.13 ± 0.18 c	3.19 ± 0.29 c
	(3*Z*)‐3‐nonen‐1‐ol	3.49 ± 0.27 a	2.70 ± 0.17 b	2.35 ± 0.25 c	2.10 ± 0.08 d
	Phenethyl alcohol	3.12 ± 0.12 a	4.16 ± 0.21 b	4.12 ± 0.28 b	4.27 ± 0.76 b
	2‐methyl‐1‐propanol	1.01 ± 0.06	–b	–	–
	Hexyl alcohol	0.95 ± 0.08	–	–	–
	3‐methyl‐1‐butanol	0.19 ± 0.01	–	–	–
	Geraniol	0.26 ± 0.02	–	–	–
	2‐octanol	1.44 ± 0.17 a	–	0.70 ± 0.02 b	–
	1‐tridecanol	0.82 ± 0.18 a	3.00 ± 0.24 b	2.83 ± 0.33 c	3.71 ± 0.39 c
	1‐tetradecanol	0.48 ± 0.15 a	2.50 ± 0.21 b	0.34 ± 0.02 c	2.38 ± 0.21b
	1‐pentadecanol	–	–	0.58 ± 0.02	–
	Subtotal	16.2 ± 1.72 a	16.0 ± 1.19 a	14.1 ± 1.27 b	15.7 ± 0.95 a
Aldehyde	Nonanal	8.62 ± 0.48 a	9.32 ± 1.45 a	9.37 ± 1.20 a	7.95 ± 0.84 b
	(*E,Z*)‐2,6‐nonadienal	26.3 ± 3.16 a	21.7 ± 1.89 b	23.1 ± 2.58 c	24.4 ± 2.21 c
	(*E*)‐2‐nonenal	10.3 ± 0.17 a	7.64 ± 0.91 b	9.41 ± 1.25 a	10.1 ± 0.36 a
	Decanal	6.20 ± 0.35 a	6.20 ± 0.20 a	6.10 ± 1.33 a	5.83 ± 0.67 b
	2‐hexenal(*E*)	4.41 ± 0.23 a	3.50 ± 0.17 b	4.30 ± 0.38 a	3.74 ± 0.48 b
	(*E*)‐hept‐2‐enal	1.25 ± 0.11	–	–	–
	Octanal	5.32 ± 0.28 a	2.60 ± 0.21 b	3.29 ± 0.17 c	5.13 ± 0.17 a
	(*E*)‐2‐octenal	0.85 ± 0.08 a	–	–	0.53 ± 0.09 b
	(*E*)‐2‐dodecenal	7.80 ± 0.87 a	5.70 ± 0.30 b	6.10 ± 0.58 c	6.34 ± 0.89 c
	Tridecanal	–	1.59 ± 0.21 a	–	0.78 ± 0.21 b
	Tetradecanal	–	0.67 ± 0.03 a	0.67 ± 0.04 a	–
	Pentadecanal	0.59 ± 0.02 a	–	0.91 ± 0.15 b	–
	Subtotal	71.7 ± 4.21 a	58.9 ± 6.68 b	63.2 ± 5.48 b	64.8 ± 5.71 b
Ketone	3‐hydroxy‐2‐butanone	1.50 ± 0.09	–	–	–
	6‐methyl‐5‐hepten‐2‐one	4.05 ± 0.14 a	5.26 ± 1.36 b	3.01 ± 1.07 c	3.09 ± 1.07 c
	6,10‐dimethyl‐5,9‐undecadien‐2‐one	2.89 ± 0.64 a	6.38 ± 1.89 b	5.32 ± 2.34 c	5.84 ± 0.75 c
	2‐tetradecanone	–	1.08 ± 0.10 a	–	0.37 ± 0.05 b
	2‐pentadecanone	0.34 ± 0.07 a	2.11 ± 0.25 b	2.51 ± 0.38 b	1.52 ± 0.11 c
	Subtotal	8.78 ± 0.71 a	14.8 ± 1.84 b	10.8 ± 2.01 c	10.8 ± 1.21 c
Ester	Isopropyl palmitate	–	0.90 ± 0.07 a	0.47 ± 0.15 b	0.99 ± 0.24 a
	Allyl hexanoate	–	–	2.99 ± 0.64	–
	Diisobutyl phthalate	0.27 ± 0.01 a	2.52 ± 0.17 b	2.56 ± 0.27 b	2.30 ± 0.19 c
	Octyl formate	–	–	0.22 ± 0.03	–
	Subtotal	0.27 ± 0.01 a	3.42 ± 0.15 b	6.24 ± 0.79 c	3.29 ± 0.33 b
Others	2‐methyl butyric acid	–	2.52 ± 0.21	–	–
	2‐pentylfuran	3.14 ± 0.32 a	4.35 ± 0.40 b	5.64 ± 1.16 c	5.43 ± 1.20 c
	Subtotal	3.14 ± 0.32 a	6.87 ± 0.57 b	5.64 ± 1.16 c	5.43 ± 1.20 c

a Data are means ± standard deviation (*n* ≥ 3). Means in the same line with a different letter represent a significant difference (*p *<* *.05).

b Not detected.

The C9 alcohol and C9 aldehyde compounds present the typical aroma of the watermelon (Tang, He, Liu, & Zhao, 2012; Yajima, Sakakibara, Ide, Yanai, & Kayashi, 1985). Specifically, the C9 alcohol included the (*E*)‐2‐nonenol and (3*Z*)‐3‐nonen‐1‐ol, and their content was ranged from 14.1% to 16.2%. The C9 aldehyde included the nonanal, (*E*)‐2‐nonenal, and (*E,Z*)‐2,6‐nonadienal, and their content was ranged from 58.9% to 71.7% (Table 1). Consequently, the C9 aldehyde content was 3.6 times of the C9 alcohol content in the watermelon juice at least. The threshold of the aldehyde is usually higher than that of the corresponding alcohol (Speirs et al., 1998). Thus, the aroma vitality of the C9 aldehyde would be 3.6 time at least of that of the C9 alcohol. Hence, the C9 aldehyde contributed 78.3% of the aroma of the watermelon juice at least.

Among the C9 aldehyde compounds, the (*E,Z*)‐2,6‐nonadienal presents an odor of the watermelon (Tang et al., 2012; Yajima et al., 1985). The threshold of the (*E*,*Z*)‐2,6‐nonadienal and (*E*)‐2‐nonenal is 0.01 and 0.08 ng/ml (Palma‐Harris, McFeeters, & Fleming, 2002; Van Boelens & Gemert, 1996), respectively. The content of the (*E*,*Z*)‐2,6‐nonadienal and (*E*)‐2‐nonenal was 26.3% and 10.3%, respectively. Consequently, the aroma vitality of the (*E*,*Z*)‐2,6‐nonadienal was 20.4 time of that of the (*E*)‐2‐nonenal. In summary, the (*E*,*Z*)‐2,6‐nonadienal was the key volatile responding to the watermelon odors in the watermelon juice.

### 3.5 Effect of UHT on formation of the aroma of the watermelon juice

**Figure 4:**
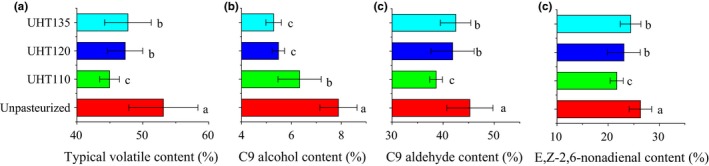
Effect of UHT on typical volatile content (a), C9 alcohol content (b) and C9 aldehyde content (c), and (*E,Z*)‐2,6‐nonadienal content (d) of the watermelon juice. C9 alcohol includes the (*E*)‐2‐nonenol and (3*Z*)‐3‐nonen‐1‐ol; C9 aldehyde includes the nonanal, (*E*)‐2‐nonenal, and (*E,Z*)‐2,6‐nonadienal

The volatiles are usually formed as the results of the heat reduction and the enzymatic metabolism during the thermal treatments (Fallik, Archbold, Hamilton‐Kemp, Loughrin, & Collins, 1997; Sanz, Olias, & Perez, 1996). The C9 alcohol and C9 aldehyde were designated as the typical volatiles. The typical volatile content of the UHT110, UHT120, and UHT135 was significantly lower than that of the Unpasteurized (Figure 4). The typical volatile reduction in the UHT110, UHT120, and UHT135 was 15.4%, 10.9%, and 10.1%, respectively. This reduction mainly came from the heat reduction. Moreover, the typical volatile content of the UHT120 and UHT135 was statistically similar, but being higher than that of the UHT110 significantly. The temperature higher than 120°C showed the similar capacity to maintain the typical volatile content of the watermelon juice. Therefore, the temperature of 120 and 135°C reduced the typical volatile content for about 10% due to the heat reduction.

On the other hand, the enzymatic metabolism is also contributed the formation of the volatiles (Sanz et al., 1996). The C9 aldehydes, especially the (*E*,*Z*)‐2,6‐nonadienal, present the typical aroma of the watermelon (Tang et al., 2012; Yajima et al., 1985), which are the metabolites of the LOX pathway. In the Lox pathway, the LOX initiates the degradation of oleic acid or linoleic acid and forms both 9‐ and 13‐hydroperoxides. Hydroperoxide lyase then cleaves the hydroperoxides to form C9 aldehydes. And then, the ADH acts to reduce the aldehydes to their corresponding alcohols. Finally, the alcohols are esterified at the present of the alcohol acetyltransferase (Bhuiya, Haque, Pradhan, & Das, 2017; Cai, 1997; Oey, Lille, Van Loey, & Hendrickx, 2008; Zhang et al., 2014). In this pathway, the increase in the LOX activity or/and the decrease in the ADH activity would benefit the accumulation of the C9 aldehyde compounds.

**Figure 5:**
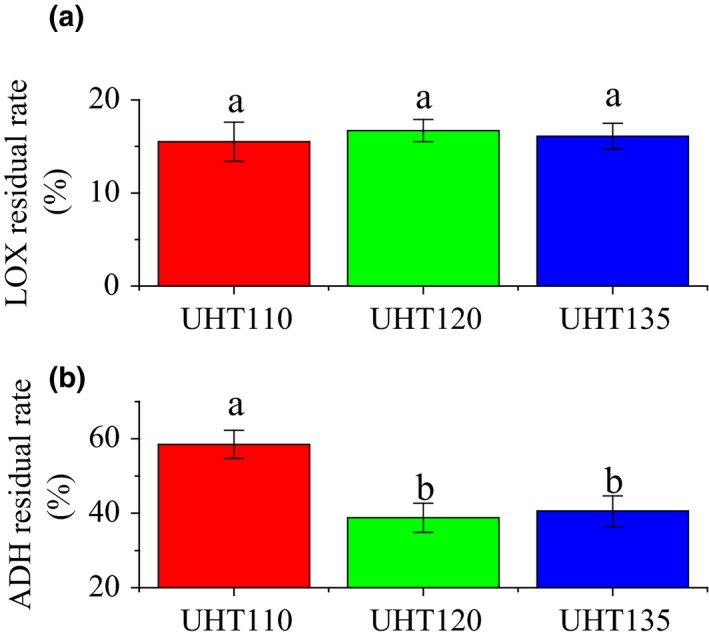
Effect of UHT on the LOX residual rate (a) and ADH residual rate (b) of the watermelon juice. LOX, lipoxygenase; ADH, alcohol dehydrogenase

The residual rate of the LOX and ADH was compared in Figure 5. The LOX activity of each temperature was reduced to about 15% of its original activity. The UHT showed no significant different on the LOX activity of the watermelon juice. Consequently, the accumulation of the C9 aldehyde content was not affected by the temperature of the UHT treatment. Being higher than our results, the LOX activity of the cucumber juice is reduced to 58.2% after the thermal treatment of 85°C for 15 s (Zhao et al., 2013). The ADH residual rate of the UHT110 was about two times of that of the UHT120 and UHT135. Consequently, the C9 aldehydes of the UHT110 would be reuded to their corresponding alcohols quickly, thereby leading to the accumulation of the C9 alcohol. Therefore, the higher ADH activity led to an accumulation of the C9 alcohol of the UHT110.

In succession, the C9 alcohol content of the UHT110 was significantly higher than that of the UHT120 and UHT135, while the C9 aldehyde content was significantly lower than that of the UHT120 and UHT135. Meanwhile, the (*E,Z*)‐2,6‐nonadienal content of the UHT110 was significantly lower than that of the UHT120 and UHT135. The (*E,Z*)‐2,6‐nonadienal content showed the similar trend to the C9 aldehyde content. The changes of the C9 aldehyde content and (*E,Z*)‐2,6‐nonadienal content were all accords with the deduction of the ADH results. The higher ADH activity of the UHT110 was responded to its low C9 aldehyde or/and (*E,Z*)‐2,6‐nonadienal content and high C9 alcohol content.

In summary, the aroma of the watermelon juice was formed by the heat reduction as well as the enzymetical metabolism. The temperature of 120 and 135°C was better to maintain the C9 aldehyde or/and (*E,Z*)‐2,6‐nonadienal content.

### 3.6 Effect of UHT on aroma of the watermelon juice

**Figure 6:**
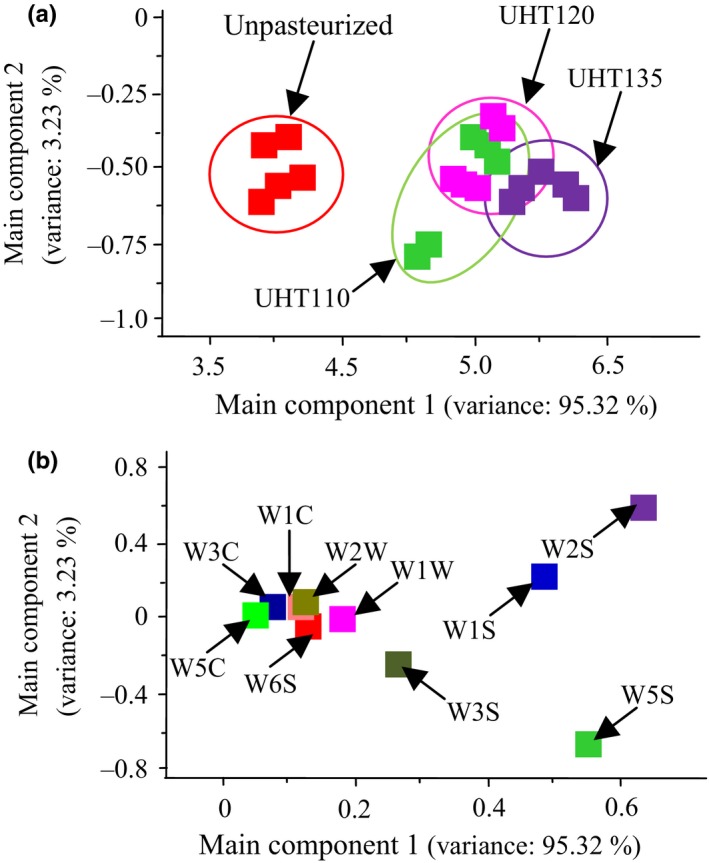
Principal component analysis (a) and loading analysis (b) of the watermelon juice

The aroma of the pasteurized watermelon juice was analyzed by the principal component analysis and loading analysis, respectively (Figure 6). Specifically, the aroma of the juice was collected by the 10 electrodes array of the electric nose. The dimensions of electrical response were reduced by the principal component analysis. The main component 1 and 2 contributed 95.32% and 3.27% for the watermelon aroma, respectively. The sum of the main component 1 and 2 reached 98.59%, contributing the main portion the aroma of the watermelon juice. Specifically, the aroma of the Unpasteurized was significantly different to that of the UHT110, UHT120, and UHT135. The aroma of each pasteurized watermelon juice was similar. Consequently, the aroma of the watermelon juice was not affected by the UHT.

The loading analysis presented the contribution of each electrode on the main component 1 and main component 2. Each electrode added positive contribution on the main component 1, and the W2S, W5S, and W1S provided the main contribution for the main component 1. The W2S is sensitive to the broad alcohol, aldehyde, and ketone, the W5S is sensitive to a broad range and especially nitrogen and oxygen compounds, and the W3S is sensitive to the methane–alkane compounds (Mannul of the Pen3). Consequently, the alcohol, aldehyde, and ketone, and methane–alkane compounds were the main contribution to the main component 1. These results were agreed with that the aldehyde and ketone were the main components in the watermelon juice by the GC‐MS analysis, which constituted 80.4% in the total volatiles. On the other hand, the W1W, W2W, W1C, W3C, W5C, and W6S gave a small influence on the main component 2. The W2S and W1S added the positive contribution on the main component 2, while the W3S and W5S added the negative contribution. The W1S is sensitive to the methyl compounds (Mannul of the Pen3). Hence, the methyl volatiles were also an important part for the aroma of the watermelon juice. These results were accorded to the results of the GC‐MS analysis.

## 4 CONCLUSION

The UHT of 120 and 135°C inactivated the microbial colonies and maintained the original color of the watermelon juice. The temperature of 120 and 135°C was also maintained the phenolic content by reducing the polyphenoloxidase activity. Moreover, the C9 aldehydes, especially the (*E*,*Z*)‐2,6‐nonadienal, presented the main aroma of the watermelon juice. The C9 aldehydes were formed as the results of the heat reduction and enzymatic metabolism. The temperature of 120 and 135°C reduced the ADH activity and well maintained the C9 aldehyde content of the watermelon juice. Hence, the temperature of 120°C of the UHT treatment was the optimal temperature for the production of the watermelon juice.

## CONFLICT OF INTEREST

None declared..
